# Supplementary material for: Single reach plans in dorsal premotor cortex during a two-target task
Source: Nat Commun. 2018 Sep 3;9:3556. doi: 10.1038/s41467-018-05959-y (PMC6120937; doi:10.1038/s41467-018-05959-y)
Supplement: Supplementary file 1 — Supplementary Information [file 41467_2018_5959_MOESM1_ESM.pdf]

Single reach plans in dorsal premotor cortex during a two-target task

Dekleva, et al.

Supplementary Note 1: Primary motor cortex

The neural results presented in the main text use only the recordings from dorsal premotor cortex. However, we also recorded from primary motor cortex for all but one session (array failure; Monkey M, session 3). The 1-Target responses were similar between areas, however M1 showed (as expected) less tuned activity during movement planning compared to movement execution (Supplementary Figure 1a). Additionally, histograms resulting from the unbiased guessing control analysis did not indicate any significant presence of dual encoding in M1 (Supplementary Figure 1b, very weak rightward skew). On a single-trial level, we saw no substantial differences between the direction of the reach plan decoded separately from PMd and M1. The single-trial decode traces obtained from M1 over all left/right 2-Target trials on the example session (Supplementary Figure 1c) were similar to those from PMd (Fig 7a). This was also true for Free-Choice trials from the same session (Supplementary Figure 1d compared to Fig 8a). To summarize these similarities, we calculated the average ΔProximity separately from PMd and M1 activity during the Target Blank epoch of each trial. While the ΔProximity magnitudes from M1 were generally closer to zero (likely due to the lower prevalence of early planning-related activity in M1), both areas largely indicated the same planned reach direction (76% - monkey C, 72% - monkey M; Supplementary Figure 1e). We did not observe any instances in which the two brain areas strongly indicated reach plans in opposite directions. Likely due to the smaller ΔProximity magnitudes during planning, M1 was less predictive of the reach direction on Free-Choice trials (72% - monkey C, 67% - monkey M; Supplementary Figure 1f) compared to PMd (80% - monkey C, 92% - monkey M). However, although M1 was in general less useful in decoding the progression of reach planning, the high degree of agreement between brain areas provides validation for the decoded planning results provided in the main text.

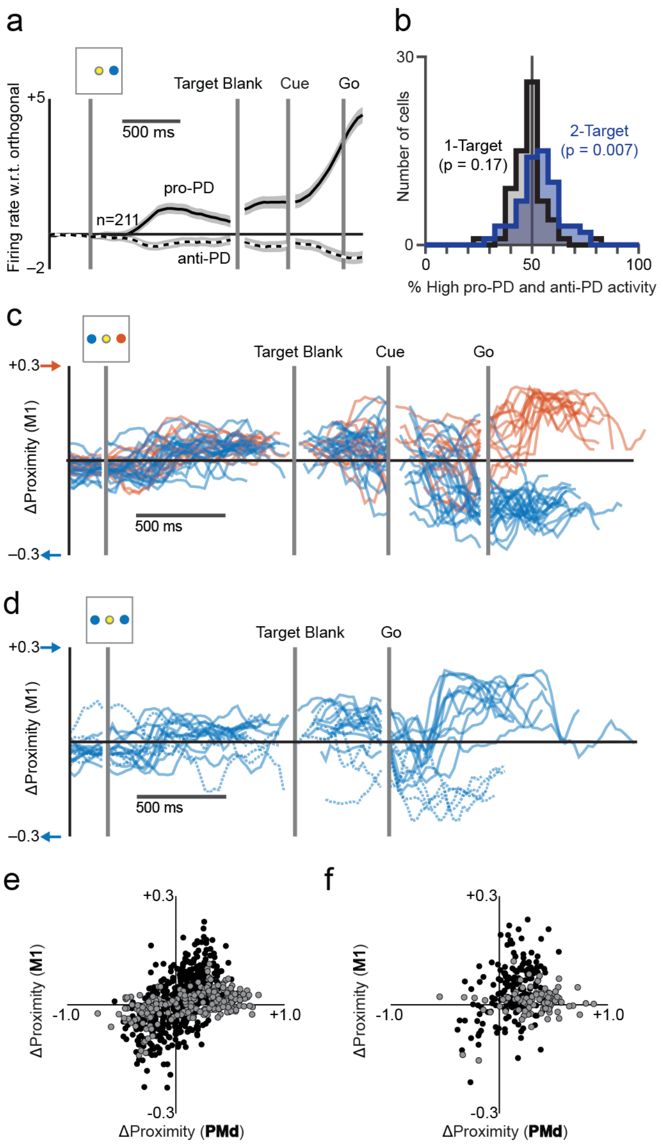

Supplementary Figure 1 | Primary motor cortex responses and comparison to PMd (a) Average activity traces of M1 neurons with preferred directions aligned (pro-PD; solid) and anti-aligned (anti-PD; dashed) with cue direction across all 1-Target trials. Activity of neurons with preferred directions orthogonal to cue direction has been subtracted. Shading represents bootstrapped 95% confidence of the mean. **(b)** Histograms showing the percentage of activity during pro-PD and anti-PD trials that exceeded the median activity observed on orthogonal-PD trials. **(c)** ΔProximity traces for all left/right trials in a single session. Blue traces indicate those for which the monkey was cued to the left. Red traces indicate trials cued to the right. **(d)** ΔProximity traces for all left/right Free-Choice trials in the example session from ***c***. **(e)** Average ΔProximity (*Target Blank* epoch) calculated from PMd and M1 activity across all cued 2-Target trials. Black markers correspond to monkey C, gray to monkey M. **(f)** as in ***e*** for all Free-Choice trials.

Supplementary Note 2: Neural response types

The similarity between PMd and M1 population responses (Supplementary Figure 1e,f) and the presence of only single reach plans—in disagreement with previous reports—raises the question of whether our PMd array placements were adequately displaced from primary motor cortex. We aimed to address this concern by characterizing single-neuron across the recorded areas. The limited size of the arrays (recorded area 4mm x 4mm) made a full categorization of the PMd-M1 gradient impossible. However, to get some sense of the cortical regions covered, we first split the PMd arrays into anterior and posterior halves (Supplementary Figure 2a,b; top). For each array region (PMd-anterior, PMd-posterior, and M1) we classified neural responses into three groups: Potential, Selected, and Movement. "Potential neurons" displayed bimodal tuning during the Target On epoch, potentially indicative of dual representation of potential targets. "Selected neurons" did not display bimodal tuning during the Target On epoch, but did exhibit unimodal tuning during the Cue epoch. Finally, "Movement neurons" exhibited tuned responses only following the Go cue. We determined whether each tuning response was significant according to the bootstrapping procedure outlined in the Methods section of the main text. Neurons that did not fall cleanly into any of these three groups were excluded. The stacked bar plots in Supplementary Figure 2a,b show the resulting distribution of response types across array regions for monkeys C and M, respectively. The response characteristics for monkey C showed a clear gradient along the anterior-posterior dimension, with a lower prevalence of Potential neurons in posterior regions. The anterior and posterior regions of the PMd array for monkey M did not exhibit any clear difference in response type, but both were clearly different (with more Potential neurons) than M1. These results confirm the anatomical observation that the PMd array for monkey M was placed further anterior than for monkey C.

The single-neuron response results from Supplementary Figure 2a,b suggest that the PMd arrays sampled from sufficiently rostral, “non-motor” regions of cortex to be distinct from M1. However, to ensure that the main results presented in the text were robust across response types, we reran the population analyses from Figures 5 and 6 after restricting our population to only Potential neurons in PMd. Supplementary Figure 2c shows the resulting scatterplot of Proximities during the Target Blank epoch of all 2-Target trials for monkey C (monkey M shown in Supplementary Figure 2d). To quantify these results, we calculated the Dual Representation Index (DRI) for the actual population activity and for the two simulated dual representation models, as in Figure 6. For both monkeys, the results strongly argued for the presence of only single reach plans (Supplementary Figure 2e,f). Thus, despite restricting our population analysis to neurons whose trial-averaged responses indicated dual representation, we still only found evidence of single reach plans on individual trials.

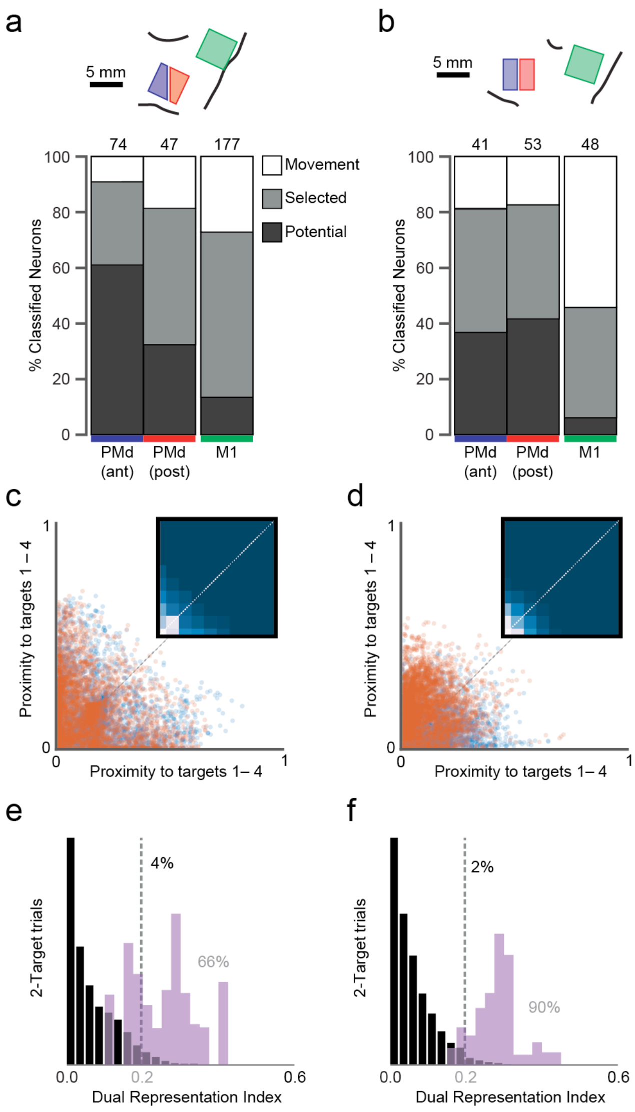

Supplementary Figure 2 | Response types across cortical regions and their effect on population-based results (a) Top: separation of array regions into PMd-anterior (blue), PMd-posterior (red), and M1 (green). Bottom: distribution of response types within each array region. Numbers above each bar indicate the total number of classified neurons. Monkey C **(b)** Same as ***a*** for monkey M. **(c)** Proximity plots of planning activity (Target Blank epoch) using only Potential neurons in PMd for all 2-Target trials as in Figure 5, monkey C. **(d)** Same as ***c*** for monkey M. **(e)** Histogram of Dual Representation Index (DRI) values using only Potential neurons in PMd for all 2-Target trials (black), monkey C. Purple bars reflect simulations from both the dual representation model and the averaged plan model, using only populations of Potential neurons. **(f)** Same as ***e*** for monkey M.

Supplementary Note 3: Standard classification versus Proximity

To evaluate the low-dimensional, neural state space activity, we devised a Proximity metric that calculates a normalized distance to known states (i.e., those observed during 1-Target reaches). We took this approach in part because it could provide a clear signature of dual-target representation if it were present (see simulation results in Fig 4). When attempting to decode a plan to either target A or B (or both) we calculated two distinct Proximities: one to the training set of 1-Target reach plans to target A, and the other to target B. As shown in the main results, single-target representations corresponded to one high proximity value and one low proximity value. Dual target representations (assessed with two models of single-neuron responses) instead resulted in two high proximities (Fig 4). This difference allowed for a simple test of dual-target decoding, as shown in Figure 6.

When decoding the temporal progression of the reach plan throughout a trial, we chose to use the difference in the two proximities from our novel classification method (ΔProximity) rather than a traditional classifier. We chose this approach because it provided a more conservative readout of the instantaneous reach plan. That is, the ΔProximity value was very small (i.e., suggesting no identifiable reach plan) unless the neural state was very close to a specific reach direction cluster identified from 1-Target data. As an illustration of ΔProximity's conservative nature compared to a traditional classifier, consider the two arbitrary, labeled clusters in Supplementary Figure 3a,b (black and white point clouds) and a test point (red point). Traditional classification methods (in this case a naïve Bayes classifier) confidently assign the point to the black cluster (Supplementary Figure 3a) while ΔProximity returned a null classification (ΔProximity = 0). Thus, ΔProximity is less likely to return a non-zero value if the neural state does not closely resemble any neural state in the training set.

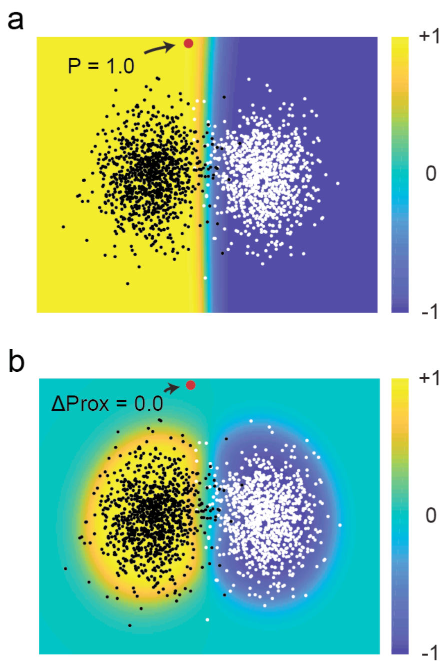

Supplementary Figure 3 | Classification using a probabilistic classifier and ΔProximity (a) Heat map of the output from a naïve Bayes classifier trained on the black and white point clouds. A test point (red dot) is confidently assigned to the left (black) group **(b)** Same as in ***a*** for the ΔProximity classifier. The test point (red) is not classified as belonging to either group.

To further demonstrate the conservative response of the ΔProximity metric to test points poorly described by the training data, we conducted a simple sensitivity analysis. First, we classified 1-Target, Target Blank neural data using both a traditional naïve Bayes classifier (Supplementary Figure 4a) and the ΔProximity classifier (Supplementary Figure 4b). Both successfully classified the correct target directions on almost all trials. We then constructed a second, artificial test set of data that made application of the classifiers inappropriate. To do this, we took the same 1-Target dataset and attempted to classify with respect to target pairs on the orthogonal axes. For example, we attempted to classify neural responses during upward 1-Target trials as either left or right reach plans, etc. The resulting outputs of the naïve Bayes and ΔProximity classifiers across trials are shown in Supplementary Figure 4c and 4d, respectively. The naïve Bayes classifier returned highly confident values indicating both left and right reach plans (peaks at -1 and +1). The ΔProximity classifier, on the other hand, returned values close

to zero, correctly indicating that no reach plans existed for either of the tested target directions. As with the toy example in Supplementary Figure 3, these results demonstrate that ΔProximity is less likely to return inappropriate or spurious decodes of the reach plan than standard classification methods.

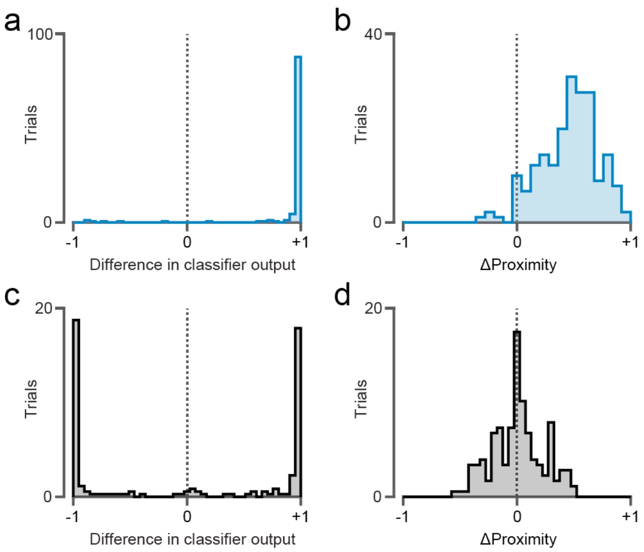

Supplementary Figure 4 | Sensitivity of naïve Bayes and ΔProximity classifiers to inappropriate data (a) Histogram of the difference in probabilities returned by a naïve Bayes classifier on 1-Target activity from an example session (Target Blank). **(b)** Same as in ***a*** for the ΔProximity classifier. **(c)** Same as ***a*** for a misaligned 1-Target dataset in which the activity corresponds to a target orthogonal to the classification axis **(b)** Same as ***c*** for the ΔProximity classifier

Supplementary Discussion

The identification of Potential neurons in PMd appears to contradict the main population-based results, which indicate only single reach plans. We suggest that the bimodal tuning that defines Potential neurons is simply an artifact caused by averaging over trials for which many have been mislabeled with the opposite reach direction. To calculate a directional tuning curve requires knowing both the direction of the reach and the corresponding neural response. However, attempting to fit tuning curves to activity that substantially precedes movement can be problematic. The direction of the planned movement—as reflected in the neural activity—might not match that of the eventual, observable movement. Either a spontaneous change of mind or a strategy based on switching plans (as we found evidence for here) can lead to spurious bimodal tuning curves. Thus, the Potential neuron responses we observed likely do not reflect actual dual representation, but simply the presence of directional tuning at a time in the trial early enough to suffer from inadvertent mislabeling of the planned direction. The absence of Potential neurons in M1 likewise stems not from a fundamental difference in function (dual representation vs. single representation), but rather just a lack of early-epoch tuning. M1 neurons display tuning generally around the time of movement execution, at which point there is little chance of a change in plan and therefore little chance of misinterpreting the represented direction.
